# Early Abnormal Placentation and Evidence of Vascular Endothelial Growth Factor System Dysregulation at the Feto-Maternal Interface After Periconceptional Alcohol Consumption

**DOI:** 10.3389/fphys.2021.815760

**Published:** 2022-02-02

**Authors:** Gisela Soledad Gualdoni, Patricia Verónica Jacobo, Camila Barril, Martín Ricardo Ventureira, Elisa Cebral

**Affiliations:** Laboratorio de Reproducción y Fisiología Materno-Embrionaria, Instituto de Biodiversidad y Biología Experimental y Aplicada (IBBEA), Consejo Nacional de Investigaciones Científicas y Tecnológicas (CONICET), Departamento de Biodiversidad y Biología Experimental (DBBE), Facultad de Ciencias Exactas y Naturales, Universidad de Buenos Aires, Buenos Aires, Argentina

**Keywords:** placenta, perigestational alcohol, vascular abnormality, VEGF system, mouse

## Abstract

Adequate placentation, placental tissue remodeling and vascularization is essential for the success of gestation and optimal fetal growth. Recently, it was suggested that abnormal placenta induced by maternal alcohol consumption may participate in fetal growth restriction and relevant clinical manifestations of the Fetal Alcohol Spectrum Disorders (FASD). Particularly, periconceptional alcohol consumption up to early gestation can alter placentation and angiogenesis that persists in pregnancy beyond the exposure period. Experimental evidence suggests that abnormal placenta following maternal alcohol intake is associated with insufficient vascularization and defective trophoblast development, growth and function in early gestation. Accumulated data indicate that impaired vascular endothelial growth factor (VEGF) system, including their downstream effectors, the nitric oxide (NO) and metalloproteinases (MMPs), is a pivotal spatio-temporal altered mechanism underlying the early placental vascular alterations induced by maternal alcohol consumption. In this review we propose that the periconceptional alcohol intake up to early organogenesis (first trimester) alters the VEGF-NO-MMPs system in trophoblastic-decidual tissues, generating imbalances in the trophoblastic proliferation/apoptosis, insufficient trophoblastic development, differentiation and migration, deficient labyrinthine vascularization, and uncompleted remodelation and transformation of decidual spiral arterioles. Consequently, abnormal placenta with insufficiency blood perfusion, vasoconstriction and reduced labyrinthine blood exchange can be generated. Herein, we review emerging knowledge of abnormal placenta linked to pregnancy complications and FASD produced by gestational alcohol ingestion and provide evidence of the early abnormal placental angiogenesis-vascularization and growth associated to decidual-trophoblastic dysregulation of VEGF system after periconceptional alcohol consumption up to mid-gestation, in a mouse model.

## Introduction

Normal growth and survival of the fetus depends on the adequate placentation. Besides providing sufficient amounts of nutrients and oxygen, the placenta establishes a privileged immune environment for fetal growth by orchestrating maternal adaptations to pregnancy and acting as a selective and protective barrier to prevent feto-maternal diseases (Creeth and John, 2020). Poor placentation and placental failure compromises fetal development leading to potential chronic diseases in adult (Sharma et al., 2016; Perez-Garcia et al., 2018; Woods et al., 2018). Experimental studies suggest that prenatal alcohol exposure disrupts the placenta (Carter et al., 2016), which may play a crucial etiologic role in alcohol-related fetal effects throughout pregnancy (Hoyme et al., 2016).

Maternal alcohol consumption can lead to the irreversible condition of fetal alcohol syndrome (FAS), the most severe form of the alcohol spectrum disorders (FASDs) (Guerri et al., 2009; de Sanctis et al., 2011; May and Gossage, 2011; Memo et al., 2013; Joya et al., 2015; Van Heertum and Rossi, 2017). FASD is characterized by fetal and birth anomalies, intrauterine growth retardation (IUGR), numerous physical, cognitive, and behavioral defects in newborns and children (Hoyme et al., 2016; Roozen et al., 2016). Prevalence of FASD was estimated at 1–30/1,000 live births in the United States (May et al., 2009; Lange et al., 2017; Popova et al., 2017), and of annual pregnancies, about 40% of women drink some alcoholic beverage during pregnancy while 3–5% of women drink heavily throughout pregnancy (Heller and Burd, 2013). In some Latin-American countries like Uruguay and Argentina, the prevalence of heavy alcohol consumption during pregnancy ranges at 40 to 75% (Magri et al., 2007; López, 2013; López et al., 2015). Despite public efforts to reduce prevalence of alcohol consumption, still high proportion of women often continues to drink moderate levels of alcohol (200 ml/day of wine containing ethanol 11%) during the early pregnancy while unaware they are pregnant (4 to 6 weeks after conception during early organogenesis) (Colvin et al., 2007). In this relation, recently we established a mouse model of perigestational moderate alcohol ingestion, previous and up to early gestation, to study the embryo developmental effects compatible with FASD. Perigestational alcohol intake up to organogenesis (equivalent to the first three-four weeks of human pregnancy) induces delayed embryo differentiation and growth, and dysmorphogenesis, by altering molecular pathways, genotoxicity, apoptosis and oxidative stress (OS) (Cebral et al., 2007, 2011; Coll et al., 2011, 2017). However, despite the direct effects of ethanol exposure on embryo-fetal outcomes, placental injury due to maternal alcohol ingestion was recently proposed as an indirect cause of fetal abnormalities and FASD (Gupta et al., 2016). Maternal alcohol-induced dysfunctional placenta was linked to IUGR, congenital defects, adulthood obesity, metabolic syndromes, cardiovascular disease (Davis et al., 2012a,b; Zhu et al., 2015; Linask and Han, 2016) and fetal programming diseases (Bada et al., 2005; Burd et al., 2007; Gundogan et al., 2008, 2015; Patra et al., 2011; Bosco and Diaz, 2012; Davis-Anderson et al., 2017; Tai et al., 2017). Nevertheless, the etiology of abnormal placenta associated to maternal alcohol consumption is proposed to be related to gestational windows of susceptibility: peri-implantation, gastrulation and/or organogenesis (first trimester in human) (Livy et al., 2004). Both early alcohol and acetaldehyde exposure may contribute to the pathogenesis of FASD by reducing placental growth and function on the first trimester (Lui et al., 2014). Particularly, perigestational moderate alcohol consumption up to peri-implantation prevents blastocyst implantation and results in early pregnancy loss (Perez-Tito et al., 2014). Since maternal alcohol exposure can decrease the trophoblast migration/invasion leading to abnormal placental vascularization (Han et al., 2012), the link between placental vasculopathy and failure in the early decidual-trophoblast development and dysregulation of angiogenesis-vascularization after perigestational alcohol ingestion up to organogenesis recently began to be studied in mouse models (Coll et al., 2018; Ventureira et al., 2019; Gualdoni G. S. et al., 2021).

At present different animal models provide insights into the alcohol-induced mechanisms on the placenta. Nevertheless, the impact of perigestational alcohol ingestion up to early gestation on placental angiogenic mechanisms involved in abnormal placenta has not been sufficiently clarified. Herein, we first provide a brief background on the gestational alcohol placental defects as the etiology of FASD, and then we extend the revision to the knowledge of emerging evidences on the effects of periconceptional consumption up to early organogenesis on placental development, highlighting the role of the trophoblast-decidual VEGF system in a mouse model.

## Main Effects of Gestational Alcohol Consumption on Placenta

Normal placental vascular development is critical for optimal fetal growth, maintenance and successful pregnancy, and subsequent life course (Adamson et al., 2002; Reynolds et al., 2006). The placenta, the major organ determinant of intrauterine growth, is involved in nutrient transport and metabolism of several molecules (Martín-Estal et al., 2019), and in the synthesis and releases of hormones and other mediators into both maternal and fetal circulations (Guttmacher et al., 2014). Alcohol use throughout gestation can disrupt the normal hormonal interactions between mother and fetus, altering natural homeostasis and hence leading to poor pregnancy outcomes. Gestational alcohol exposure impaires the respiratory gases supply due to poor placental vascularity, leading to hypoxia, thus resulting in pregnancy complications, IUGR, and preeclampsia, malnutrition, or stillbirth (Aliyu et al., 2008, 2011; Salihu et al., 2011; Carter et al., 2016). Ethanol interferes with placental transport of nutrients, oxygen, and waste products (Gundogan et al., 2008, 2015; Kwan et al., 2020). Moreover, prenatal alcohol exposure alters the placental iron transport yielding to fetal iron deficiency anemia, condition that exacerbates alcohol-related growth restriction (Kwan et al., 2020). Also, animal and *in vitro* studies have suggested that chronic and heavy alcohol use in pregnancy may impair transport of folic acid across the placenta to the fetus by decreasing expression of transport proteins, thus contributing to the deficits observed in FASD (Hutson et al., 2012). In overall, alcohol use throughout pregnancy promotes poor fetal outcomes and relevant clinical manifestations of FASD by inducing abnormal placental morphogenesis and impairment of placental metabolism and hormonal function (Gundogan et al., 2008; Ramadoss and Magness, 2012c; Gupta et al., 2016).

Gestational alcohol intake produces the “alcohol-related placental associated syndrome” (Salihu et al., 2011) that includes miscarriage, hypertension, preeclampsia, preterm birth, placenta previa, placenta accreta and placental hemorrhage (Gundogan et al., 2010; Meyer-Leu et al., 2011; Avalos et al., 2014; Carter et al., 2016; Tai et al., 2017; Ohira et al., 2019; Orzabal et al., 2019; Odendaal et al., 2020). Moreover, high risk of placental abruption was observed after consumption of 7–21 drinks per week (a mean of two drinks per day and BAC of 5–100 mg/dL) (Burd et al., 2007). In human and animal models, alcohol exposure during pregnancy usually reduces placental weight and size, affecting directly the structure and function (Gundogan et al., 2008, 2015; Bosco and Diaz, 2012; Carter et al., 2016; Kwan et al., 2020). The most generalized effects of gestational alcohol intake on the placenta were on its vasculature, which is associated with uteroplacental malperfusion, resistance, and placental and umbilical cord contraction (Iveli et al., 2007). In mouse models, alcohol exposure at mid-gestation leads to severe embryo-placental growth retardation (Haycock and Ramsay, 2009).

Gundogan et al. (2008, 2010) reported, in animal model, that one major placental abnormality due to chronic gestational ethanol exposure is the failure of maternal decidual spiral artery remodeling by which the interaction between the invasive trophoblasts and maternal vessels is impared and leads to altered placental blood flow and nutrient exchange. Moderate or high-dose of ethanol intake during gestation also reduces the labyrinthine development (Gundogan et al., 2008, 2010, 2013, 2015). During third trimester of gestation, alcohol affects the uteroplacental vascular function (Rosenberg et al., 2010; Subramanian et al., 2014; Orzabal et al., 2019) by impairment of uterine spiral artery remodeling, angiogenesis and vasodilation (Radek et al., 2005), *via* altered endothelial angiogenic gene expression (Ramadoss and Magness, 2012b) and proteome defects (Ramadoss et al., 2011; Ramadoss and Magness, 2012c). Table 1 summarized the main relevant findings, on the placental effects produced by gestational alcohol ingestion in human and/or murine models.

**TABLE 1:** Summary of the main findings on the placental effects produced by gestational alcohol ingestion.

Gestational period of alcohol ingestion	Model	Alcohol intake pattern	Placental effects	References
Along gestation	Human	Moderate quantities	Reduced placental weight	Burd and Hofer, 2008
			Impaired blood flow/artery vasodilatation	Ramadoss and Magness, 2012b,c
			Abnormal nutrient transport	Gundogan et al., 2015
			Fetal resorption, miscarriage	
			Umbilical cord vasoconstriction, IUGR	
Along gestation	Human	High alcohol quantities	Growth restriction, fetal hypoxia	Gundogan et al., 2008, 2010, 2015
			Reduced blood flow and nutrient interchange	
			Fetal hypoxia, IUGR	
Along gestation	Human	Two drinks (wine)/day (BAC 5–100 mg/dL)	Placental abruption, IUGR, FAS	Burd et al., 2007
		(18–30 g ethanol/day)	Abnormal fetus	
Along gestation, or during:	Human	Heavy, moderate and/or light drinking	Utero-placental malperfusion and hypoplasia	Tai et al., 2017
2nd, 3rd, 1st + 3rd or 2nd + 3rd trimesters			Premature delivery, IUGR	
Gestational days 7–17	Rat	Ethanol 4.5 g/kg/day (BAC 216 mg/dL)	Uterine vascular disfunction	Ramadoss and Magness, 2012c
				Subramanian et al., 2014
One occasion across gestation	Human	Binge-heavy (8 drinks on 1.5 days/week)	Decreased placental growth	Carter et al., 2016
Along gestation (days 6–18)	Mouse	BAC 110 mg/dL	Placental resistance,	Ramadoss and Magness, 2012a
			Abnormal vascular perfusion	
Gestational days 6–16	Rat	18-24-37% EDC	Incompleted uterine vascular transformation	Gundogan et al., 2008, 2010, 2015
Along gestation	Human	High quantities- severe intake	Vascular resistance, vasoconstriction	Siler-Khodr et al., 2000
				Acevedo et al., 2001
				Reynolds et al., 2006
				Ramadoss and Magness, 2011
				Bosco and Diaz, 2012
Chronic binge-like during gestation	Human		Impaired maternal uterine artery reactivity	Subramanian et al., 2014
			Vascular dysfunction	
			Decreased uterine vasodilation	
Pre-conception until early gestational	Primate	1.5 g/kg/day of a 4% ethanol	Reduced vascular perfusion in late placenta	Lo et al., 2017
		(=6 drinks/day)	Altered fetal vasculature in late placenta	
Gestational day 8.75	Mouse	Acute (two i.p injections 3 g/kg ethanol)	Reduced late placental labyrinth	Haghighi Poodeh et al., 2012
			Altered cell junctions of placental barrier	
			Increased permeability	

*BAC, blood alcohol concentration; i.p., intraperitoneal; EDC, ethanol derived calories; IUGR, intrauterine growth restriction.*

Adequate mutual interactions between decidual and trophoblast tissues during early placentation determine the normal vascularization of the placenta at term. Placental defective growth and angiogenesis, linked to altered early maternal vascular remodeling and trophoblast invasion (Woods et al., 2017, 2018), may cause pregnancy failure, placental insufficiency, preeclampsia, fetal developmental disorders, and preterm birth (Cha et al., 2012). Advances in the understanding of placental abnormalities and the main mechanisms involved induced by maternal alcohol exposure are usually studied in the mouse model of placentation (Probyn et al., 2012), which briefly is given below.

## Overview of Mouse Placental Development and Vascularization as a Model for Placental-Alcohol Effect Studies. Role of the Vascular Endothelial Growth Factor System

In mouse, placentation begins with implantation (days 4.5–6 of gestation), when blastocyst’s mural trophectodermal cells invade the uterine epithelium (Woods et al., 2018). After implantation, placental development goes through the gestational phases of gastrulation (days 6 to 8.5), organogenesis (days 9 to 11.5) and the fetal phase (days 12–19).

### Decidual Development and Maternal Vascularization

Decidualization and maternal angiogenesis are pivotal to provide normal placental vascularization at term. Immediately with implantation, antimesometrial uterine stromal cells proliferate and transform in decidual cells, forming the avascular and densely packed decidual tissue (day 6 of gestation). Following decidualization extends toward mesometrial region of the implantation site (days 7.5–10), transformation of uterine spiral arteries occurs by angiogenesis (Das, 2009, 2010). The smooth muscle layer of spiral arteries close to the trophoblastic zone, normally largely remodel and disappear and thus, profound mesometrial decidual vascular growth takes place to allow uterine vascular elongation and provide maternal oxygenated blood flow to the fetal face of placenta (Cross et al., 2002; Kim et al., 2013; Ventureira et al., 2019). Maternal angiogenesis and vasodilation, involving also endothelial proliferation, are promoted mainly by decidual and uterine natural killer cells (uNKs) (Blois et al., 2011; Sojka et al., 2019). The uNKs have vital roles in decidual vascular remodeling and dilation of spiral arteries (Charalambous et al., 2012; Hofmann et al., 2014; Lima et al., 2014), in trophoblast invasion control (Ashkar and Croy, 2001; Ashkar et al., 2003; Croy et al., 2003; Felker and Croy, 2017; Meyer et al., 2017a,b), decidualization, and immune tolerance (Greenwood et al., 2000; Wallace et al., 2012; Rätsep et al., 2015). Mouse strains genetically ablated from uNK cells, not only fail to undergo smooth muscle spiral artery remodeling but also show abnormal branching of the vascular bed, leading to implantation sites with anomalous vascularization (Yougbaré et al., 2017). The VEGF of uNK cells is necessary to guide the maternal angiogenesis (Gargett et al., 2001; Wulff et al., 2002; Zygmunt et al., 2003; Heryanto et al., 2004; Taylor, 2004; Coultas et al., 2005).

### Development of Placental-Trophoblast Layer

After implantation, in mouse gestational days 7–8.5, differentiated polyploid invasive primary trophoblast giant cells (TGCs) first invade the maternal microvasculature around the conceptus (Malassine et al., 2003), while the polar trophectoderm forms the extra-embryonic ectoderm and, from it, the chorionic cells and the ectoplacental cone (EPC). The allantois grows out, attaches and fuses to the chorionic trophoblast forming the chorio-allantoic placenta. Meanwhile, secondary TGC cells differentiate at the margin of the EPC, and become invasive to remodel mesometrially the maternal extracellular matrix (ECM) and microvasculature (Woods et al., 2018).

At organogenesis, the mouse placenta consists of three layers: the decidua, the junctional zone (JZ), and the labyrinth. The JZ, the outer layer limiting the decidua, is constituted by spongiotrophoblast cells (SpT), and the invasive cells: TGCs (P-TGC), spiral artery-associated TGCs (SpA-TGCs) and glycogen cells (GC) (Malassine et al., 2003; Woods et al., 2018). The labyrinth develops when the attached allantoic mesoderm invaginates and interdigitates into the chorion, to form the extensive vascular fetal network of definitive placenta. The labyrinth is composed of the fetal vessels, chorionic-mononuclear trophoblast cells and the allantoic mesenchyme. While the fetal vessels undergo extensive branching, the chorionic trophoblasts differentiate into sinusoidal-TGCs and the syncytiotrophoblast cells develop; all togheter form, with the fetal endothelium, the interhemal barrier (Watson and Cross, 2005; Hu and Cross, 2010; Rai and Cross, 2014; Woods et al., 2018).

When the definitive placental vascularization is established (around day 11 of gestation), major trophoblast invasion to maternal spiral arteries begins. While TGCs remodel the decidual ECM (interstitial invasion) (Knöfler et al., 2001; Rai and Cross, 2014), spiral artery-TGCs replace the endothelial cells of dilated spiral arteries (endovascular invasion). This maternal vessel transformation leads to distended, high flow and low-resistance maternal sinusoids at JZ (Cross et al., 2002), where the maternal blood is funneled in trophoblast-lined conduits in absence of arteriolar vasoconstriction. These vascular remodeling processes directed by trophoblast are major key for successful progression of pregnancy. Abnormal invasion (sub- or over-invasiveness) and maternal endothelial remodeling/replacement are key etiologic mechanisms associated to placental pathologies and pregnancy complications (Perez-Garcia et al., 2018). Placental angiogenesis-vascularization occurs under production and control of growth and vasoactive factors, such as the vascular endothelial growth factor (VEGF) (Hess et al., 2007).

### Vascular Endothelial Growth Factor System During Placentation

**FIGURE 1:**
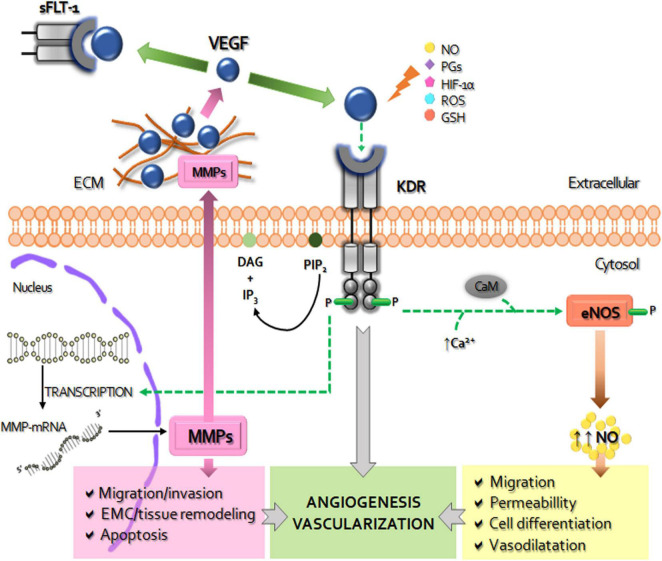
Schematic diagram of the role of VEGF system in the placentation. The vascular endothelial growth factor (VEGF) activates the membrane kinase insert domain receptor (KDR) by phosphorylation, triggering specific signaling cascades. By increasing intracellular calcium (Ca^++^) and calmodulin (CaM) VEGF-KDR binding induces the expression and activity of nitric oxide synthase (NOS) to produce increase of nitric oxide (NO), a strong inducer of increased vascular permeability, vasodilation, cell migration and differentiation. The other *via* of VEGF-KDR activation, related to fosfatidil inositol 4,5-bisfosfato (PIP-2) and diacylglycerol (DAG), leads to the transcription of metalloproteinases (MMPs). MMPs are secreted to the extracellular matrix (ECM) where, among other functions, degrade and remodel the ECM and release the ECM-sequestered VEGF by cleaving proteins that retain it in the matrix. The soluble receptor tyrosine kinase similar to fms-1 (sFLT-1) can prevent the binding of VEGF to KDR, thus decreasing it pro-angiogenic and survival activity. VEGF expression can be modified by NO, prostaglandins (PGs), hypoxia inducible factor (HIF-1α), reactive oxygen species (ROS), reduced glutathione (GSH), among other factors. In overall, placental activated VEGF system mediates the angiogenesis-vascularization during placentation.

The VEGF system is involved in angiogenesis-vascularization during placentation (Figure 1). VEGF is expressed in arteriolar smooth muscle, endothelium, in trophoblast, uNKs and decidual cells (Hoffmann et al., 2007; Zhang et al., 2011; Hofmann et al., 2014; Li et al., 2014; Ventureira et al., 2019). Its expression is regulated by hypoxia through transcription of the hypoxia inducible factor (HIF)-1α (Maltepe et al., 2005). During early gestation, HIF-1α regulates the trophoblast proliferation and differentiation during EPC development (Takeda et al., 2006). A hypoxic-oxidant environment induces the synthesis and release of anti-angiogenic factors, leading to failures in trophoblastic differentiation, invasion and placental angiogenesis (Perez-Garcia et al., 2018; Woods et al., 2018).

Vascular Endothelial Growth Factor acts through binding to three receptors with intrinsic tyrosine kinase activity: VEGF-R1 (FLT-1), VEGF-R2 (KDR/Flk-1) and VEGF-R3 (FLT-3) (Kimura and Esumi, 2003). Main physiological effects of VEGF are attributed to KDR, whereas FLT-1 modulates VEGF signaling *via* ligand sequestration (Chung and Ferrara, 2011). KDR activation induces cell proliferation and migration (Lara et al., 2018), permeability (Yancopoulos et al., 2000), decidualization, trophoblastic differentiation and invasion, and nutrient uptake of the placenta (Reynolds and Redmer, 2001; Haghighi Poodeh et al., 2012). In pathological placentas, VEGF is reduced whereas the soluble form of FLT-1, responsible for the endothelial dysfunction, is increased (Roberts and Escudero, 2012; Li et al., 2014).

The VEGF-KDR binding activates signaling cascades that stimulate the production of at least 11 angiogenic factors (Apte et al., 2019), such as the endothelial nitric oxide synthase (eNOS) and matrix metalloproteinases (MMPs). The nitric oxide (NO), produced by oxidation of L-arginine, is an important regulator of the placental vasodilatation, participating in vascular smooth muscle relaxing, the increase of blood flow, and reduction of platelet aggregation and thrombosis (Krause et al., 2011).

The other major contributors to VEGF-mediated angiogenesis are the MMPs. These multigenic proteolytic zinc-dependent enzymes are composed by six classes (collagenases, gelatinases, stromelysins, matrilysins, membrane-type MMPs, and other MMPs) (Amălinei et al., 2007; Cui et al., 2017; Henriet and Emonard, 2019). MMPs are involved in proliferation, apoptosis, migration, differentiation, tissue and ECM remodeling, protein degradation (Lemaître and D’Armiento, 2006; Hamutoğlu et al., 2020), and trophoblast survival and invasion (Isaka et al., 2003; Agaoglu et al., 2016). MMP-2 (gelatinase A) and MMP-9 (gelatinase B) play a role in endometrial tissue remodeling at implantation (Novaro et al., 2002), in decidualization (Fontana et al., 2012), in trophoblast invasiveness (Staun-Ram et al., 2004; Plaks et al., 2013; Espino et al., 2017; Gualdoni G. et al., 2021), in endothelial cell morphogenesis (Chandrasekar et al., 2000). Expansion of the uterus to accommodate the growing embryo and the maternal vascular establishment depend on MMP-2 and -9 (Sternlicht and Werb, 2001). At early mouse gestation, TGCs are positive for MMP-2 and MMP-9 expression (Alexander et al., 1996; Bany et al., 2000; Bai et al., 2005), while during organogenesis mainly MMP-9 participates in TGC-invasion and labyrinthine vascularization (Fontana et al., 2012; Gualdoni G. et al., 2021; Gualdoni G. S. et al., 2021). VEGF up-regulates the MMP-2 and MMP-9 expression in human umbilical vein endothelial cells (Heo et al., 2010). Imbalances of VEGF system and subsequent alterations in MMP-2 and MMP-9 expression-activity take relevance in abnormal placentation (Gualdoni G. S. et al., 2021) and in various placentopathies (Amălinei et al., 2007; Cui et al., 2017).

## Pathophysiologic Mechanisms Involved in Alcohol-Associated Placental Abnormalities and Role of the Angiogenic Vascular Endothelial Growth Factor System

The alcohol-induced abnormal placental mechanisms are multifactorial. The alcohol dispersed into placenta is primary detoxified by other placental systems (CYP2E1) different from the alcohol dehydrogenase (ADH) and aldehyde dehydrogenase (ALDH) enzymes (Gemma et al., 2007), because of their low affinity/activity in placental tissues (Heller and Burd, 2013). By this, gestational alcohol exposure impacts on the metabolism of placenta producing oxidative stress (OS) (Kay et al., 2000; Gundogan et al., 2008, 2010, 2015). Although antioxidant enzymatic and glutathione activities have been shown in the placenta after gestational alcohol ingestion (Qanungo and Mukherjea, 2000), OS impacts strongly on trophoblastic function and leads to pregnancy loss (Gundogan et al., 2010). Perigestational moderate alcohol ingestion up to organogenesis in mouse produces protein nitration, lipid peroxidation and DNA damage in trophoblast-decidual tissue (Coll et al., 2018). As a result of alcohol-induced OS and decreased bioavailability of NO, insufficient early placental vascularization and arteriolar vasoconstriction can cause placental hypoxia (Acevedo et al., 2001; Wareing et al., 2006; Gualdoni G. S. et al., 2021).

Stimulatory and inhibitory effects of ethanol on VEGF have been reported, depending on alcohol administration patterns (Radek et al., 2008; Haghighi Poodeh et al., 2012; Jegou et al., 2012). In chick extraembryonic tissues, moderate and heavy alcohol exposure for 24 or 48 h, impaires vascular development and downregulates VEGF and its receptors (Gu et al., 2001; Tufan and Satiroglu-Tufan, 2003). Together with enhanced permeability and altered placental barrier, VEGF was up-regulated in the CD-1 mouse placenta at 9.5–14.5 days of gestation after two 4-h interval intraperitoneal doses of 3 g/kg ethanol injected 8.75 days post-coitum (Haghighi Poodeh et al., 2012). Ethanol treatment decreases VEGF in yolk sac membranes by inhibition of angiogenic genes due to excess of alcohol-induced reactive oxygen species production (Wang et al., 2016). However, diminished placental vascular density after early alcohol exposure significantly decreased KDR expression in placenta at term (Holbrook et al., 2019). In addition, in alcohol-induced hypoxic placenta, the release of anti-angiogenic soluble receptor sFlt-1 (Reyes et al., 2012), is associated with maternal endothelial dysfunction (Roberts and Cooper, 2001; Roberts et al., 2011). Thus, alterations in the VEGF-VEGF-R, caused by oxidative stress, may be the main cause important imbalances in placental angiogenesis induced by alcohol.

The VEGF downstream molecular expression, such as NO and MMPs, may be altered by alcohol. Chronic binge-like alcohol decreases uterine arterial endothelial eNOS expression in an animal model of third trimester-equivalent of human pregnancy (Ramadoss et al., 2011), in a similar way as does the acute ethanol exposure in human placenta. However, chronic and acute ethanol exposure seems to induce eNOS activity in the fetoplacental unit in other model and HUVEC cells, respectively (Acevedo et al., 2001). Anyway, inhibition or stimulation of NO synthesis by alcohol use throughout pregnancy leads to vasoconstriction of the placenta and umbilical vessels and results in hypoxia and reduced fetal malnutrition (Lui et al., 2014; Gundogan et al., 2015; Lo et al., 2017; Holbrook et al., 2019; Ohira et al., 2019). Consequently, the impaired placental function leads to an increase in oxidative stress that compromises placentation as it alters trophoblast cell motility (Kay et al., 2000; Gundogan et al., 2008). Also, gestational alcohol exposure can alter transcription of MMPs and affects the maternal uterine vascular remodeling (Orzabal et al., 2019).

## Perigestational Alcohol Consumption Up to Early Gestation: Effects on Mouse Placentation and Role of the Vascular Endothelial Growth Factor System

**FIGURE 2:**
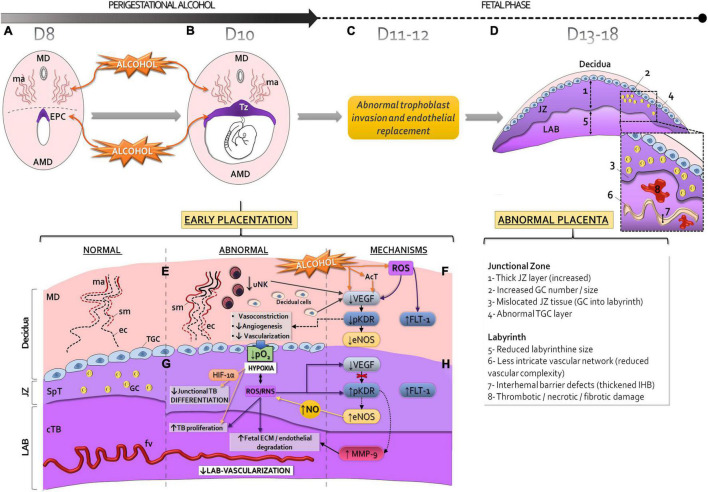
Proposed mechanisms of abnormal early placentation and vascularization and long-term impact to placenta produced by perigestational alcohol consumption, in the mouse model. Perigestational alcohol (OH) intake up to gastrulation-organogenesis [day 8 (D8) **(A)** and 10 (D10) **(B)**, disrupts the maternal spiral artery (ma) remodeling of mesometrial decidua (MD), and alters the ectoplacental cone (EPC) **(A)** and subsequent development of trophoblastic zone (Tz) of the early placenta **(B)**. Consequently, after cessation of alcohol ingestion (day 11, D11), abnormal trophoblast invasion and endothelial replacement may occur **(C)**, leading to abnormal structure and function of the placenta at term **(D)**. The placental abnormalities can be originated in altered angiogenesis-vascularization of decidual-trophoblastic tissues **(E,G)** during perigestational alcohol exposure **(B)**. Alcohol and/or its metabolites [acetaldehyde, AcT, reactive oxygen species (ROS)] impact on the early placentation processes, including changes in uNK cells. Consequently, diminished junctional trophoblast differentiation and low labyrinthine growth and vascularization can occur **(G)**. The decidual-trophoblastic abnormalities can be associated with altered endothelial nitric oxide synthase (eNOS) and metalloproteinases (MMP)-2 and/or MMP-9 expression/activity due to abnormal vascular endothelial growth factor (VEGF) expression and KDR activation (pKDR) or increased FLT-1 **(F,H)**. The disrupted VEGF-receptor-NOS-MMPs mechanisms are linked to hypoxia and increased HIF-1α, and reactive oxygen species and/or reactive nitrogen species (ROS/RNS) production. Sm, smooth muscle cells of spiral arteries; ec, endothelial cells; cTB: chorionic trophoblastic cells; SpT, spongiotrophoblastic cells; GC, glycogen cells; TGC, trophoblast giant cells; fv, fetal vessels; Lab, labyrinth; JZ, junctional zone; TB, trophoblast; ECM, extracellular matrix; AMD, antimesometrial decidua.

The origins of uteroplacental insufficiency and vasculopathy at late gestation may be caused by abnormal placentation during early pregnancy, including the peri-implantation period (Burton and Jauniaux, 2010; Kajantie et al., 2010). Evidence is currently lacking, in animal models, to explain the effects and cellular-molecular pathways responsible for late placental abnormalities induced by perigestational alcohol consumption (PAC) until early gestation (Gårdebjer et al., 2014; Kalisch-Smith and Moritz, 2017; Kalisch-Smith et al., 2019). Here, in a mouse model, we propose that PAC up to organogenesis disrupts the early decidual-trophoblastic development and vascularization (Figures 2A,B; Perez-Tito et al., 2014; Coll et al., 2018; Ventureira et al., 2019; Gualdoni G. S. et al., 2021), leading to incomplete maternal vascular remodeling due to trophoblast invasion defects (Figure 2C), and later, to abnormal placenta (Figure 2D).

After PAC up to day 10 of gestation, the lumen expansion of decidual spiral arteries is reduced (Figure 2E; Ventureira et al., 2019), producing a poor dilation of maternal vascular bed. Alcohol could alter the decidual artery endothelial organization and reduces cell proliferation. PAC disrupts the arterial smooth muscle cell remodeling and leads to permanent muscle wall in decidual vessels. These defects could be associated to decreased number of uNK cells in decidua, which may also be involved in low dilation and less branching of maternal spiral arteries (Figure 2E; Ventureira et al., 2019).

Reduced decidual angiogenesis after PAC can be explained in part by the decreased VEGF expression in decidual and uNK cells probably due to OS (Coll et al., 2018; Ventureira et al., 2019; Figure 2F). However, down-regulation of KDR expression in decidual and endothelial cells after PAC could also be involved in the abnormal decidual vascularization. FLT-1 drives anti-angiogenic effects by its binding to VEGF (Ferrara, 2004; Lima et al., 2014; Felker and Croy, 2017), and its increase was associated with oxidative factors (Kim et al., 2013). After PAC, OS may induce FLT-1 expression in decidual and uNK cells causing abnormal decidual angiogenesis (Ventureira et al., 2019; Figure 2F).

Impaired downstream VEGF signaling, due to reduced activation of KDR (pKDR) after alcohol consumption, leads to a decreased expression/activity of eNOS in the decidual endothelium, contributing to maternal artery vasoconstriction and reduced angiogenesis (Figure 2F). The immediate consequence of low decidual vascularization, because of unremodeled maternal vessels, is a deficient blood perfusion, increased blood pressure and flow velocity. Reduced tissue oxygenation increases HIF-1α expression levels in the trophoblastic interface, which is consistent with a hypoxic-oxidative state (Gualdoni G. S. et al., 2021; Figure 2G).

At organogenesis, the labyrinth is still growing in a low oxygen environment, but junctional trophoblastic cells become more differentiated and invasive with increased oxygenation (Cowden Dahl et al., 2005; Pringle et al., 2010). PAC produces trophoblastic growth deficiency and TGC and spongiotrophoblast cell abnormalities compatible with trophoblast differentiation alterations (Gualdoni G. S. et al., 2021; Figure 2G). Subsequent abnormal invasion (Figure 2C) can lead to placentopathy later (Woods et al., 2017; Figure 2D). Paralely, the PAC-induced deficient labyrinthine vasculogenesis is associated to a densely packed tissue due to increased chorionic trophoblastic cell proliferation (Gualdoni G. S. et al., 2021; Figure 2G). The early insufficient labyrinthine vascularization generates persistent hypoxia and OS and embryo growth restriction and malformations at organogenesis (Cebral et al., 2007; Coll et al., 2011, 2017; Gualdoni G. S. et al., 2021).

Although hypoxia is a strong stimulus for placental VEGF expression (Zhang et al., 2015), this expression decreased in the exposed-trophoblastic tissues (Gualdoni G. S. et al., 2021). Despite VEGF reduction, probably due to OS and/or to sequestering by FLT-1, alcohol induces high trophoblastic KDR phosphorylation (Figure 2H). Following PAC, downstream KDR activation results in increased trophoblastic eNOS expression, which over-produces NO, causing placental OS. However, eNOS expression could also be triggered by FLT-1 pathways (Bussolati et al., 2001) or induced by hypoxia since eNOS promoter contains hypoxia response elements (Schäffer et al., 2006). Once ethanol induces NO production, this factor is able to cause phosphorylation of KDR (Bussolati et al., 2001). Despite KDR activation in JZ and labyrinth, MMP-2 and MMP-9 expression decreased in the former but only MMP-9 increased in the later, resulting in tissue-dependent adverse alcohol effects in the exposed trophoblastic tissues (Gualdoni G. S. et al., 2021; Figure 2H).

In conclusion, PAC up to organogenesis leads to early abnormal placentation by defective decidual-trophoblastic development and disruption of complex angiogenic cellular processes, in which the VEGF system results one of the major affected mechanisms. PAC up to early pregnancy may lead to placental abnormalities and vasculopathy compatible with abnormal placentas associated to FASD.

## Author Contributions

EC have proposed the topic of this revision and prepared the draft of the manuscript. GG and EC edited the text. GG designed the figures. PJ, CB, and MV co-wrote the manuscript. All authors approved the last version of the manuscript.

## Conflict of Interest

The authors declare that the research was conducted in the absence of any commercial or financial relationships that could be construed as a potential conflict of interest.

## Publisher’s Note

All claims expressed in this article are solely those of the authors and do not necessarily represent those of their affiliated organizations, or those of the publisher, the editors and the reviewers. Any product that may be evaluated in this article, or claim that may be made by its manufacturer, is not guaranteed or endorsed by the publisher.
